# Computer-assisted 3D reconstruction of the human basal forebrain complex

**DOI:** 10.1590/s1980-57642008dn10200005

**Published:** 2007

**Authors:** Lea Tenenholz Grinberg, Helmut Heinsen

**Affiliations:** 1 LTG, MD, PhD, Department of Pathology of Faculty of Medical Sciences of University of Sao Paulo, Brazil; Israeli Institute of Education and Research Albert Einstein of São Paulo, Brazil.; 2 HH, MD, Prof., Labor fuer Morphologische Hirnforschung der Klinik und Poliklinik fuer Psychiatrie und Psychotherapie.

**Keywords:** human, adults, models/structural, substantia innominata, nucleus basalis of Meynert, quantitative analysis, neurons/cytology

## Abstract

The basal forebrain complex (BFC) is a small but intricate structure. Its organization and function is hard to investigate using conventional methods, especially in humans. By combining new methods of research we present a comprehensive overview of this complex, in order to better understand its function in normal and diseased brains.

**Methods:**

The right and left BFC of a 29-year-old male were reconstructed from gallcocyanin (Nissl) stained 440 µm-thick serial horizontal sections by using advanced computer-assisted 3D reconstruction software.

**Results:**

The reconstructed components in the present case include Ch2, Ch3, Ch4am-al, Ch4i, Ch4p, juxtacommissural, Ayala’s medial (subpallidal) and lateral (periputaminal) subnuclei. These components are arranged in an arch-like course mainly beneath the anterior commissure. The bilateral volume of all subnuclei was 99.06 mm^3^, the left side accounting for 48.05 mm^3^. Some of the subnuclei exhibited volume asymmetry indices varying from 28.3 to 12.9%.The volume of Ayalas’ lateral or periputaminal nucleus was 9.7% higher on the right, than on the left side.

**Conclusions:**

Our methodological approach promises to be highly efficient and reproducible in studying morphofunctional correlations in complex cognitive features

## Abstract (pt)

O complexo prosencefálico basal (CPB) é uma estrutura complicada, apesar de pequena. É difícil estudar sua organização e função por métodos convencionais, especialmente em humanos. Ao combinar novos métodos de investigação, apresentamos uma abordagem completa deste complexo, com objetivo de auxiliar a compreensão de sua função em cérebros controles e acometidos por doenças.

**Métodos:**

Os CPBs direito e esquerdo de um homem de 29 anos de idade foram reconstruídos tridimensionalmente com uso de um software potente, à partir de secções histológicas horizontais de 440 µm de espessura.

**Resultados:**

Os núcleos reconstruídos no presente caso são: Ch2, Ch3, Ch4am-al, Ch4i, Ch4p, e os subnúcleos juxtacommissural, Ayala medial (ou subpalidal) e lateral (periputaminal). Essas estruturas se arranjam em forma de arco, praticamente acompanhando a comissura anterior. O volume bilateral de todas as estruturas é de 99.06 mm^3^, sendo que o lado esquerdo responde por 48.05 mm^3^. Alguns dos subnúcleos exibem índices de assimetria variando de 28.3 a 12.9%. O volume do subnúcleo de Ayala lateral ou periputaminal é 9.7% maior à direita.

**Conclusão:**

O método apresentado tem grande potencial de ser bastante eficiente e reprodutível para estudos de correlação morfofuncional relacionadas a características cognitivas complexas.

Large chromophilic neurons of the basal forebrain complex (BFC) are the major source of cholinergic innervation to isocortical and allocortical regions as well as to subcortical nuclei. The BFC is implicated in attention, memory and learning processes.^1,2^ Classically, this complex is subdivided into four cell groups: Ch1 corresponding to the medial septal nucleus; Ch2 and Ch3 corresponding to the nucleus of vertical and horizontal limb of the diagonal band of Broca, respectively; and Ch4 also referred to as the nucleus basalis of Meynert.^3,5^ More recently, emphasis has been placed on another group of cells, the so-called Ayala's nucleus.^6,7^

The BFC is affected in several neurodegenerative disorders including Alzheimer’s disease, Parkinson’s disease, Korsakoff’s disease,^8,9^ progressive supranuclear palsy^10^ and corticobasal degeneration.^11^ Cholinesterase inhibitors intended to compensate the loss of acetylcholine in the cerebral cortex, are frequently used in current pharmacological therapies for Alzheimer’s disease

For methodological reasons, most of the investigations carried out to date have been based on animal models comprising mainly rodents and to a lesser extent, primates.^12,13^ Although these studies depict considerable details regarding neuroanatomy, connections and biochemical features, the human BFC is likely to display an even more specific degree of organization.^14^ In addition, considering the selective vulnerability of the cells of this complex shown in neurodegeneration, it is important to have a more detailed understanding of the human BFC in order to allow more specific investigations into this complex.

A 3D reconstruction of the human BFC was previously published in 2006.^7^ Currently, further data based on bilateral horizontal sections will be presented in order to provide more detailed aspects on size, shape, parcellation and asymmetry of this complex.

## Methods

The brain of a 29-year-old male, whose cause of death was pulmonary arrest, was formalin-fixed within the first 24 hours after death. The detailed procedure of fixation, dehydration, celloidin mounting and gallocyanin staining of the brain was described by Heinsen et al.^15^ In brief, the frontal, parieto-occipital and most lateral part of the temporal lobes of the present case, were severed leaving the central parts of the tel- and diencephalon intact. This central part/block was dehydrated in graded series of ethanol solutions (70, 80, 96%) for 1 week per stage and soaked in celloidin. This hardened celloidin-embedded block was sectioned horizontally on a sliding microtome at a thickness of 440 µm. Every slice was stained with gallocyanin, dehydrated, coverslipped and mounted with Permount^®^, as outlined in detail by Heinsen et al.^15^

In addition, both hemispheres of a 66-year-old male were processed in an identical manner and serially cut in the coronal plane. The brain was removed 8hrs after death and the preservation of the tissue was excellent. However, bilateral artifacts at the caudal level of the substantia innominata prevented a 3D reconstruction of the BFC in this case.

### Computer assisted 3D reconstruction

A total number of 50 consecutive gallocyanin stained sections of the central block containing all parts of the BFC were photographed with a digital SLR-camera with close-up lenses mounted. These pictures were imported into a computer-assisted 3D reconstruction program (Amira 3.1^®^, Mercury Computer Systems Inc.). Each individual section was aligned manually by means of the Amira align editor. We applied Mesulams et al. terminology on the nuclei of the BFC, i.e. Ch2 and Ch3 corresponds to the vertical and the horizontal limb of the diagonal band of Broca, respectively, and Ch4 to the basal nucleus of Meynert.^3,12,16^ Ch1, the medial septal nucleus, was not reconstructed in this case. Furthermore, we adopted the terminology proposed by Simic et al.^6^ and Boban et al.^17^ for the medial and lateral parts of Ayala’s nucleus. Finally, we favored the term anterior commissure – juxtacommissural cells (for the sake of simplicity, called just juxtacommissural) for clusters of large basophilic neurons closely arranged around the anterior commissure.^7^ Subsequently, the outlines of all subnuclear components of the BFC, as well as the profiles of both fornices and anterior commissure were identified in each gallocyanin stained section and traced manually on the digital pictures with the help of a graphic tablet. Amira^®^ converts all the outlines into digital coordinates for generating a surface based upon the individual outlines. Moreover, the in-built modules of Amira^®^ can calculate surface areas and volumes of 3D reconstructed objects.

The volumetric side differences of each subnucleus were expressed by the asymmetry index of Eidelberg and Galaburda.^18^ Negative values represent right-sided volume predominance, whereas positive values indicate the opposite.

### Results

**Figure 1:**
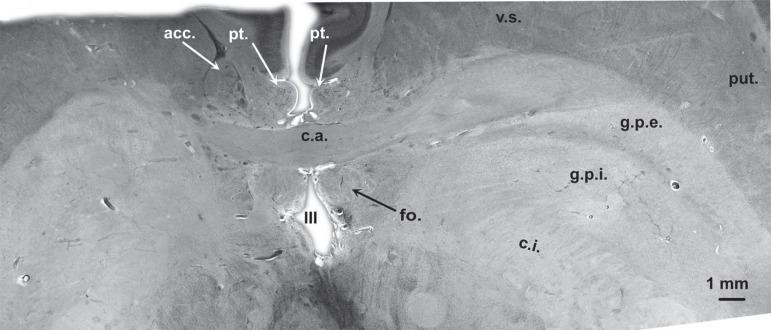
Horizontal gallocyanin stained section through the fornix and dorsomedial parts of the anterior commissure. Red line in Figure 6 indicates plane of section in Figure 1.

**Figure 2:**
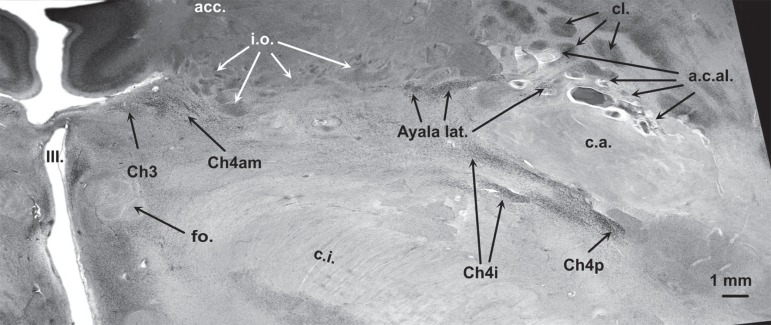
Horizontal gallocyanin stained section 4.4 mm ventral to plane of section indicated in Figure 1. Plane of section is indicated in Figure 6 by the white line.

**Figure 6:**
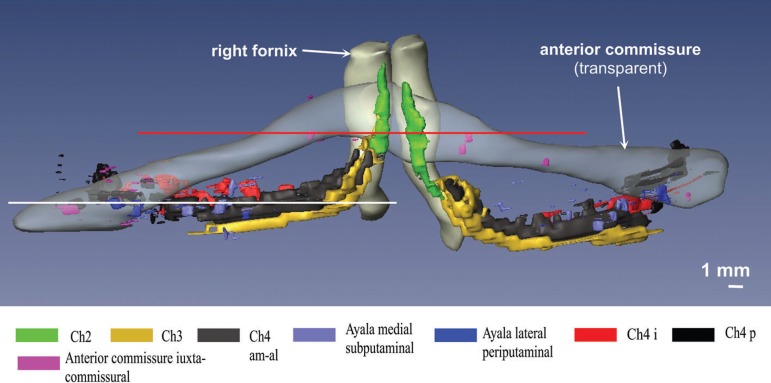
Basal forebrain complex in a fronto-occiptal perspective.

The BFC is a term that denotes a number of subcortical nuclei, coinciding externally with the region of the anterior perforated substance (APS). Its dorsal limits are formed by the striatum together with its fundus striati. On mediosagittally cut hemispheres, the anterior commissure, the paraterminal (subcallosal) gyrus and the diagonal band - which emerges in a ventrolateral course from the ventral tip of the paraterminal gyrus - are additional macroscopically visible structures belonging to the basal forebrain. Furthermore, the fornical columns and the anterior commissure are used as reference structures for the localization of BFC components. Figures 1 and 2 are from two different horizontal sections through the BFC. The plane in Figure 1 passes through the most dorsal part of the yoke-like anterior commissure (see also red line on Figure 6). Rostral to the anterior commissure, the round profiles of the paraterminal gyrus can be identified. At this plane of section the paraterminal gyri, as well as the fornical columns are descending in parallel. Both are separated by the anterior commissure. In a closer view, dispersed fine black dots representing single large multipolar neurons of the vertical limb of the diagonal band of Broca can be distinguished within the paraterminal gyrus. The plane of section is slightly inclined to the right side; therefore the profiles of the anterior commissure do not completely match. On both sides, the anterior commissure is abutting the ventral striatum rostrally and the globus pallidus laterally and caudally. The plane of section in Figure 2 is parallel to that in Figure 1, however, it is exactly 4.4 mm more ventral than the former one (see also white line in Figure 6). Mainly the right hemisphere is depicted in Figure 2 at a higher magnification than in Figure 1. The right fornical column and the lateral parts of the anterior commissure can be easily identified. At this plane of section, the paraterminal gyrus is no longer visible since its ventrolateral extension, called the horizontal limb of the diagonal band of Broca, is following another direction, traversing the surface of the anterior perforate substance. The region between the caudal rim of the ventral striatum (or nucleus accumbens at this plane of section) and the ventral parts of the internal capsule, as well as the regions lateral to the fornix or medial to the outlines of the anterior commissure is called substantia innominata.

The substantia innominata comprises: conspicuous cell islands mainly composed of small-sized neurons (i.e., olfactory islands), nuclei of the lateral hypothalamus that are cell-sparse and difficult to delineate, and irregular aggregates of rather large darkly stained (chromophilic) neurons that constitute the BFC. Some of these neuronal aggregates are closely associated with perforating branches of the central anterolateral arteries (Figure 2, a.c.a.l.). At this plane of section the latter are easily identifiable by conspicuous perivascular spaces.

**Figure 3:**
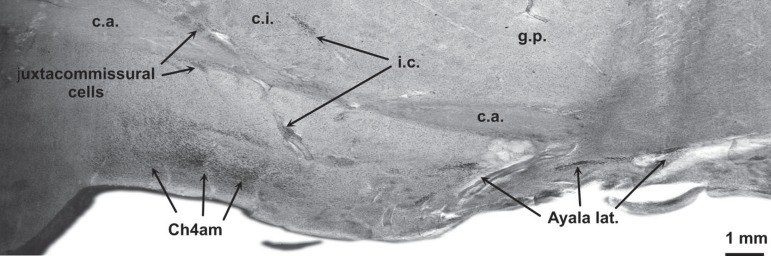
Coronal gallocyanin stained section through the right hemisphere of a 66-year-old male. Figure 1-3, gallocyanin-stained 440 ìm-thick celloidin mounted sections. **a.c.al.**, central anterolateral arteries; **acc. ncl.**, accumbens.; **Ayala lat.**, lateral or periputaminal part of Ayala’s nucleus; c.a., anterior commissure; **Ch4am**, basal ncl. of Meynert, anteromedial part; **Ch4i**, basal ncl. of Meynert, intermediate part; **Ch4p**, basal ncl. of Meynert, posterior part; **c.i.**, internal capsule; **cl**, claustrum; **fo**, fornix; **g.p.**, globus pallidus; **g.p.e.**, external globus pallidus; **g.p.i.**, internal globus pallidus; **i.c.**, interstitial cells; **III**, third ventricle; **i.o.**, olfactory islands; **pt**, paraterminal (subcallosal) gyrus; **put**, putamen; **v.p.**, ventral pallidum; **v.s.**, ventral striatum.

**Figure 4:**
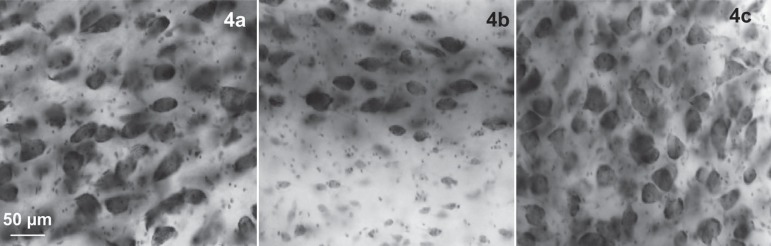
Microscopic view of large chromophilic neurons of Ch4am (A), of Ayala’s lateral or periputamenal nucleus (B), and of Ch4p (C).

The subnuclei of the BFC are parcellated according to size, shape, density, and staining characteristics of their constituent neurons and by their topography. Three examples of cytological criteria are given in Figure 4A-C. From medial to lateral, the outlines of Ch3, Ch4am, lateral or periputaminal Ayala's nucleus, Ch4i and Ch4p can be delineated in Figure 2. Juxtacommissural cells cannot be seen at both horizontal levels (Figures 1 and 2), however they can be identified in the coronal plane of section in Figure 3.

**Figure 5:**
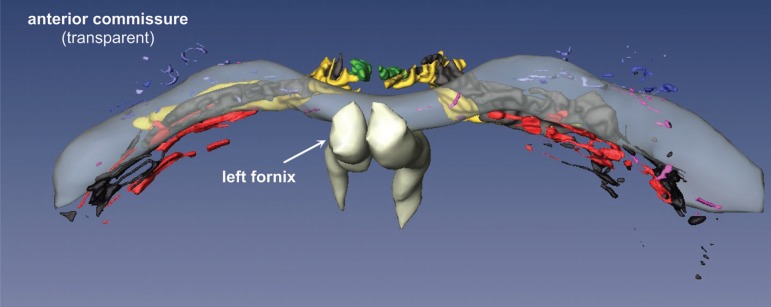
Basal forebrain complex seen from dorsal after reconstruction with Amira.

The complicated spatial arrangement of the BFC can be only perceived after a computer-assisted 3D reconstruction of the subnuclear profiles (Figures 5 and 6). In our 3D reconstruction the most rostral parts of the lateral or periputaminal Ayala’s nucleus are found about 1.5 mm frontal to the plane of the most rostral parts of Ch4am. In a dorsoventral view, the components of the BFC are mainly confined to the trajectory of the anterior commissure (Figure 5). Exceptions to this rule are Ch2 and dorsal Ch3 nuclei, as well as most of the components of Ayala's nucleus, which are located rostral to the anterior commissure. Parts of the Ch4i and Ch4p are likewise not completely covered by the caudal rim of the anterior commissure. In a rostrocaudal view (Figure 6) it is clearly recognizable that the intermediate parts of the anterior commissure and the Ch4am-al subnucleus take a disparate course. Consequently, the intermediate parts of these structures are separated by a wide cleft.

The juxtacommissural cells (Figure 3) are characterized by their close association with the anterior commissure. However, they are arranged in widely dispersed patches and do not form a continuous sheath around the internal capsule.

We have excluded from our 3D reconstruction, large basophilic neurons that cluster apart from the Ch1 to Ch4 continuum. These interstitial (12;16) or outlying cells (19) can be easily recognized in coronal sections (Figure 3, i.c.). In a 66-year-old male a perivascular aggregate of large basophilic cells proved to be continuous in serial sections with a more dorsal cell cluster at the ventrolateral side of the internal capsule.

The total volume of all components of the BFC is 99.06 mm^3^. Some of the components exhibit quite conspicuous side differences, e.g., the right juxtacommissural complex is 28.3% larger than the left one (Figure 4, Table 1). The left-sided Ch2 subnucleus is longer in the vertical axis (Figure 6) and its volume is 13.9% higher on the left, than on the right side (Table 1). The volumes of the Ch4am-al subnuclei are also asymmetric with 12.9% predominance of the right side. The asymmetry between the lateral (periputaminal) Ayala's subnuclei are less marked. In the present case, the volume of the right-sided subnucleus was larger by 9.7% (Table 1).

**Table 1:** Volumes of the subnuclear components of the BFC in mm_. Asymmetry index according to Eidelberg and Galaburda (20). A minus sign indicates a predominant rightsided volume of a nuclear complex in the BFC.

Nucleus	Side			Asymmetry index
	Left mm^3^	Side Right mm^3^	Total mm^3^	
Ayala medial	0.52	0.57	1.09	-0.023
Ayala lateral	0.25	0.37	0.62	-0.097
Pericommisural	0.10	0.36	0.46	-0.283
Ch2*	3.9	2.27	6.17	0.132
Ch3^†^	17.27	14.86	32.13	0.038
Ch4am_al^‡^	17.91	23.22	41.13	-0.129
Ch4i^§^	4.45	3.92	8.37	0.032
Ch4p^||^	3.65	5.44	9.09	0.099
Total	48.05	51.01	99.06	-0.015

* nucleus of the vertical limb of the diagonal band of Broca;

† nucleus of the horizontal limb of the diagonal band of Broca;

‡ anteromedial-anterolateral parts of the nucleus basalis of Meynert;

§ intermediate part of the nucleus basalis of Meynert pars intermedia;

|| posterior part of the nucleus basalis of Meynert.

### Discussion

The subnuclei of the BFC are subject to age-related nerve cell loss, as well as early appearance of neurofibrillary tangles in Alzheimer’s disease.^20-26^ In addition, neuronal loss in the BFC has been hypothesized to represent a common mechanism leading to dementia in some neurodegenerative diseases.^8^ Therefore, its integrity is crucial for cognition, attention, and memory.

In a recent publication, we succeeded in correlating MRI signal changes with the coordinates of the Ch4am-al–Ch4p subnuclear continuum.^27^ This kind of point-to-point MRI-neuroanatomy correlation may be a significant methodological achievement to verify the integrity of the BFC and can be used for advanced in-vivo imaging methods aiming to diagnose early Alzheimer’s disease, as well as to monitor its progression or to study the effects of new drugs to slow down or to halt progression of Alzheimer’s disease.

Our present reconstruction based on horizontal serial sections yields similar results to the previous work based on coronal serial sections.^7^ A slight difference can be detected when comparing the spatial relationship between Ch2 and Ch3. In our first reconstruction, Ch2 and Ch3 were running in parallel. However, the present reconstruction based on horizontal serial sections reveals that these subnuclei neurons are intricately interwoven. The complex arrangement of the Ch2 and Ch3 components can be better traced in horizontal sections through the human BFC.

The juxtacommisural cells were not included in this reconstruction due to their considerable interindividual variation concerning size, shape and localization. It is not clear whether Mesulam et al.^12^ were implicitly including the components of Ayala’s nucleus into their interstitial cell clusters. The rostral components of the lateral nucleus of Ayala are located conspicuously apart from the Ch4 complex. Together with its particular cytoarchitectonics features (Figure 4B) and specific connections with Broca’s area,^6^ this spatial separation would be a further argument to categorize Ayala’s nucleus as an entity.

Several authors have described asymmetries in the neuronal number of the BFC.^21,28-32^ Special focus was directed on Ayala’s nucleus whose asymmetry was explained by its cholinergic axons to Broca’s speech area.^6,17^ We succeeded in visualizing size and shape differences of the periputaminal or lateral Ayala’s subnucleus by computer-assisted 3D reconstruction.^7^ While only 3D reconstructions from the left hemisphere were available in our previous publication, both hemispheres were available for the current study. The lateral Ayala's subnucleus is an inconspicuous nuclear group compared to the other subnuclei (Figures 5 and 6). In contrast with the observations of Simic et al.^6^, the right Ayala´s nucleus was 9.7% bigger than left nucleus. On the other hand, the volume differences between the Ch2 and Ch4am-al subnuclei were far more expressed, ranging from 28.3 to 12.9% (Table 1). These asymmetries could either represent an extreme example of the well-known individual variability of the human CNS or result from uncertainties in the cytoarchitectonic parcellation of the outlines. Further studies involving a larger number of subjects are necessary to confirm these asymmetry findings. Lowes-Hummel et al.^30^ described a higher neuron number in the right BFC in their samples. Furthermore, such asymmetries could reflect size differences in the cortical projection areas.^21,22^

We were unable to find published volume data for the complete BFC in humans. However, Halliday et al.^33^ published bilateral Ch4 volumes varying from 76 to 154 mm^3^. This is in line with our present data of 58.6 mm^3^ in the case investigated. Previous authors have shown that the components of the BFC receive multivariate afferents and send their cholinergic axons to different brain regions.^2,34-36,36-41^ Therefore, considering our preliminary results, we believe it necessary to study the subnuclei of the BFC together with the regions connected to them. A combination of in-situ post-mortem MRI, computer-assisted 3D reconstructions and classical stereological analyses will help to avoid methodological errors and to correct shrinkage factors due to histological procedures. In addition, a statistical analysis of the quantitative results correlated with clinicofunctional data of the patients will facilitate unraveling the morphofunctional peculiarities of the human brain. This objective can be achieved by combining the specific facilities of the University of Sao Paulo and the Wuerzburg University in a similar way as has been documented in this publication.
